# Supplementary material for: Low-Temperature Synthesis of Cu-Doped Anatase TiO2 Nanostructures via Liquid Phase Deposition Method for Enhanced Photocatalysis
Source: Materials (Basel). 2023 Jan 9;16(2):639. doi: 10.3390/ma16020639 (PMC9862325; doi:10.3390/ma16020639)
Supplement: Supplementary file 1 [file materials-16-00639-s001.zip › materials-2110174-supplementary.pdf]

Supporting Information for:

Low-Temperature Synthesis of Cu-Doped Anatase TiO_2 Nanostructures Via Liquid Phase Deposition Method for Enhanced Photocatalysis

Mitsuhiro Honda [1,*], Tsuyoshi Ochiai [2], Popy Listiani [1], Yuma Yamaguchi [1] and Yo Ichikawa [1]

[1] Graduate school of Engineering, Nagoya Institute of Technology, Nagoya, Aichi 466-8555, Japan

[2] Materials Analysis Group, Kawasaki Technical Support Department, Kanagawa Institute of industrial Sci-ence and TEChnology (KISTEC), Kawasaki 213-0012, Japan

*Corresponding author: honda.mitsuhiro@nitech.ac.jp (M. Honda)

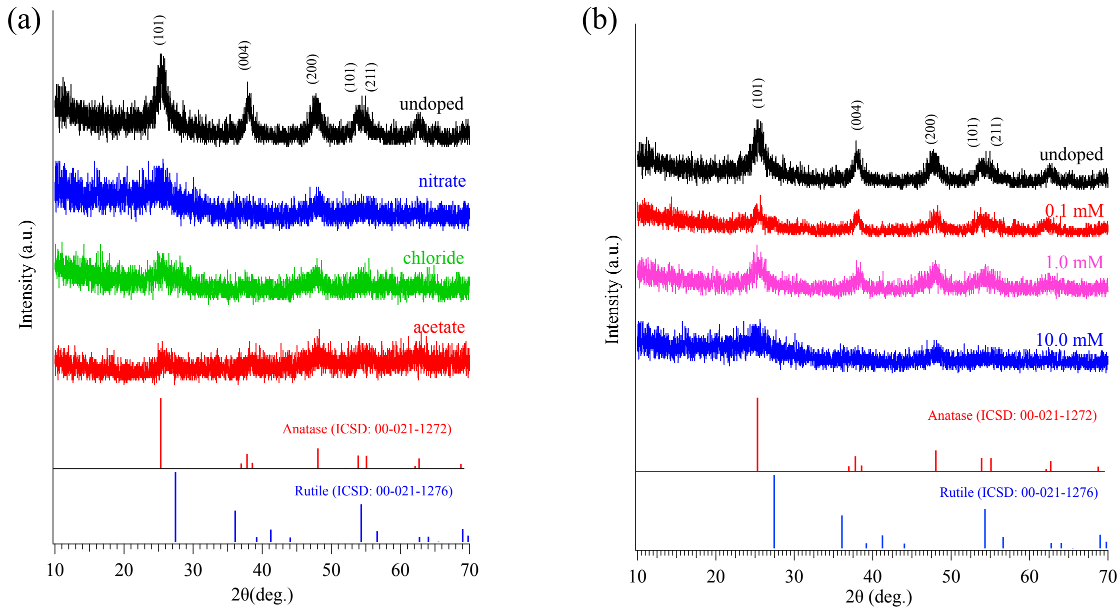

Figure S1 XRD patterns of undoped (black) and Cu-doped TiO_2 (colors). In panel (a), different copper salts, nitrate, chloride, and acetate are denoted by blue, green, and red, respectively. In panel (b), red, pink, and blue colors indicate different concentrations, 0.1, 1.0 and 10.0 mM, respectively.

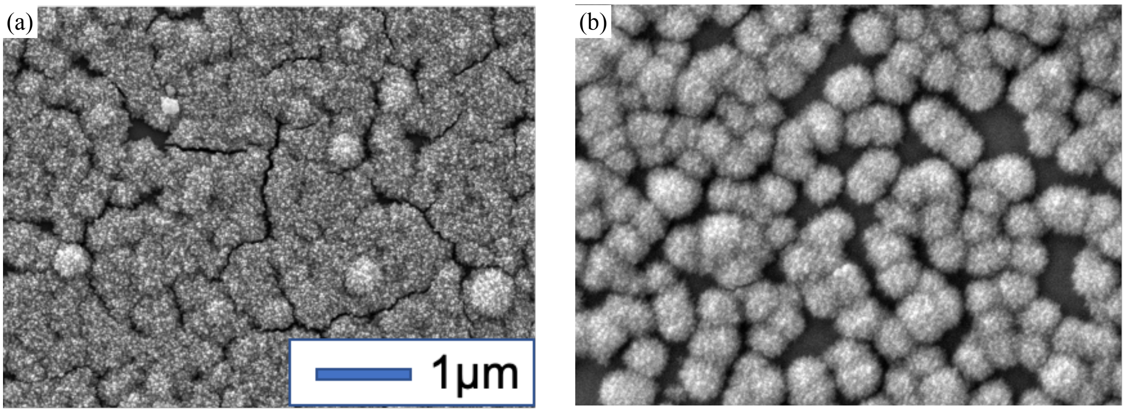

Figure S2 SEM images of TiO_2 coated on a glass substrate (a) with and (b) without hydrophilization treatment by UV illumination

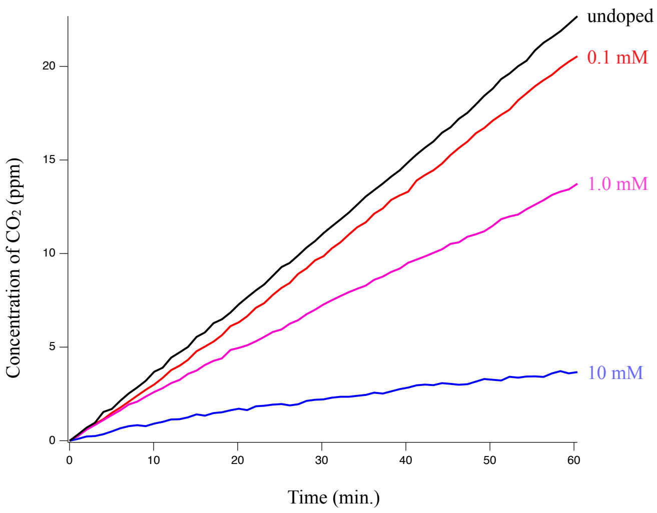

Figure S3 Production of CO_2 during the decomposition of acetaldehyde.

Table S1 Peak positions and FWHM values obtained by fitting XPS data

	Position (eV)	FWHM (eV)
Ti $2p_{1/2}$	458.4	1.6
Ti $2p_{3/2}$	464.1	2.1
Cu 2p: Cu^{+}	931.7	1.8
Cu 2p: Cu^{2+}	932.4	2.0
